# Expression profiling of tomato pre-abscission pedicels provides insights into abscission zone properties including competence to respond to abscission signals

**DOI:** 10.1186/1471-2229-13-40

**Published:** 2013-03-09

**Authors:** Toshitsugu Nakano, Masaki Fujisawa, Yoko Shima, Yasuhiro Ito

**Affiliations:** 1 Food Biotechnology Division, National Food Research Institute, NARO, 2-1-12 Kannondai, Tsukuba, Ibaraki 305-8642, Japan

**Keywords:** Tomato (*Solanum lycopersicum*), Abscission zone, Flower pedicel, Auxin, Shoot meristem, Competence to respond to abscission signals, Transcription factor, MADS-box genes

## Abstract

**Background:**

Detachment of plant organs occurs in abscission zones (AZs). During plant growth, the AZ forms, but does not develop further until the cells perceive abscission-promoting signals and initiate detachment. Upon signal perception, abscission initiates immediately; if there is no signal, abscission is not induced and the organ remains attached to the plant. However, little attention has been paid to the genes that maintain competence to respond to the abscission signal in the pre-abscission AZ. Recently, we found that the tomato (*Solanum lycopersicum*) transcription factors *BLIND* (*Bl*), *GOBLET* (*GOB*), *Lateral suppressor* (*Ls*) and a tomato *WUSCHEL* homologue (*LeWUS*) are expressed specifically in pre-abscission tissue, the anthesis pedicel AZs. To advance our understanding of abscission, here we profiled genome-wide gene expression in tomato flower pedicels at the pre-abscission stage.

**Results:**

We examined the transcriptomes of three tomato flower pedicel regions, the AZ and flanking proximal- (Prox) and distal- (Dis) regions, and identified 89 genes that were preferentially expressed in the AZ compared to both Prox and Dis. These genes included several transcription factors that regulate apical or axillary shoot meristem activity. Also, genes associated with auxin activity were regulated in a Prox-Dis region-specific manner, suggesting that a gradient of auxin exists in the pedicel. A MADS-box gene affecting floral transition was preferentially expressed in the Prox region and other MADS-box genes for floral organ identification were preferentially expressed in Dis, implying that the morphologically similar Prox and Dis regions have distinct identities. We also analyzed the expression of known regulators; in anthesis pedicels, *Bl*, *GOB*, *Ls* and *LeWUS* were expressed in the vascular cells of the AZ region. However, after an abscission signal, *Bl* was up-regulated, but *GOB*, *Ls* and *LeWUS* were down-regulated, suggesting that *Bl* may be a positive regulator of abscission, but the others may be negative regulators.

**Conclusions:**

This study reveals region-specific gene expression in tomato flower pedicels at anthesis and identifies factors that may determine the physiological properties of the pre-abscission pedicel. The region-specific transcriptional regulators and genes for auxin activity identified here may prevent flower abscission in the absence of signal or establish competence to respond to the abscission signal.

## Background

Plants can detach aged leaves, unfertilized flowers, diseased or damaged organs and mature fruits or ripe seeds. These abscission processes enable plants to recycle nutrients for continuous growth, develop appropriate organs, survive diseases, and facilitate reproduction [1,2]. Abscission occurs at predetermined positions called abscission zones (AZs). The AZ contains a group of small cells that lack large vacuoles, suggesting that these cells may be arrested in an undifferentiated state [3]. Control of abscission has been an important agricultural concern because of its substantial effect on crop yield and quality. For example, humans have selected germplasms with reduced seed shattering during the domestication of grains such as rice (*Oryza sativa*), maize (*Zea mays*), and wheat (*Triticum aestivum*) [4-6]. Also, in tomato (*Solanum lycopersicum*), “jointless” cultivars with mutations inhibiting pedicel AZ development have been widely adopted for mechanical harvesting, because in the absence of an AZ, the stem and sepals remain on the plant, allowing the fruit to be harvested without the green tissues.

Abscission can be divided into four major steps [7]: (1) development of the AZ, (2) acquisition of competence to respond to abscission-promoting signaling, (3) activation of abscission, and (4) sealing of the break by differentiation of a protective layer on the main body side of the AZ. AZ development in tomato fruit/flower pedicels has been extensively investigated and several mutations that affect pedicel AZ development have been identified. For example, *jointless* (*j*) and *jointless2* (*j2*) mutations completely suppress AZ differentiation and the *lateral suppressor* (*ls*) mutation partially impairs AZ development [1,8-10]. The *j* and *ls* loci encode a MADS-box transcription factor and a GRAS family transcription factor, respectively [11,12]. The *j2* locus remains to be identified but is predicted to encode a C-terminal domain (CTD) phosphatase-like protein [13]. Recently, we determined that the MADS-box transcription factor MACROCALYX (MC), which was identified as a regulator of sepal size [14], also regulates tomato pedicel AZ development by interacting with the MADS-box protein encoded by the *j* locus [10].

In other systems, investigation of genes involved in AZ structure development (step1) identified several genes regulating the formation of the AZ. In Arabidopsis, the MADS-box transcription factor gene *SEEDSTICK* (*STK*) and the bHLH transcription factor gene *HECATE3* (*HEC3*) regulate the formation of seed AZs [15,16], and *BLADE-ON-PETIOLE1* (*BOP1*) and *BOP2*, which encode BTB/POZ domain and ankyrin repeat containing NPR1-like proteins, regulate formation of floral organ AZs [17]. In rice, pedicel AZ formation for seed shattering is regulated by *qSH1*, which is a major chromosome 1 quantitative trait locus for seed shattering and encodes a BELL-type homeobox transcription factor, and *SH4*, which is a major chromosome 4 seed shattering quantitative trait locus and encodes a MYB3 DNA-binding domain containing protein. Rice pedicel AZ formation is also regulated by *SHATTERING ABORTION1* (*SHAT1*) encoding an AP2 family transcription factor, the rice *SHATTERING1* homologue (*OsSH1*) encoding a YAB family transcription factor, and *CTD phosphatase-like protein1* (*OsCPL1*) [6,18-21].

Studies of steps 3 and 4, activation of abscission and differentiation of the protective layer, have also revealed several factors required for abscission. For example, the phytohormones auxin and ethylene stimulate abscission-promoting signaling and regulate the onset of abscission in an antagonistic fashion; auxin inhibits the onset of abscission and ethylene promotes abscission [1,2,22]. Cell wall degrading and modifying enzymes are activated in the AZ separation layers [23-26]. In addition, transcription factors, receptor-like kinases, signal peptides, chromatin remodeling factors and membrane-trafficking proteins are involved in the activation of abscission [24]. Furthermore, genome-wide transcriptome analyses revealed that a wide variety of genes for phytohormone signaling, cell wall degradation, and defense related proteins are up-regulated during abscission [27-29].

Although many studies have examined the other steps of abscission, the mechanisms that drive step 2, acquisition of competence to respond to abscission signals, have remained mysterious. Prior to the onset of abscission, AZs possess the ability to respond to abscission-initiating signaling but the AZ cells have not initiated abscission. For example, tomato pedicel AZs at anthesis remain in an idling state before receiving abscission-promoting signals. Once the signal is provided, the AZ cells immediately start abscission, but if the flower is successfully pollinated, then the AZ cells remain small, dividing as the AZ structure grows thicker to support the growing fruit. The cells in stage 2 are seemingly static and inactive, but we recently found that AZs of tomato pedicels at anthesis show a distinct gene expression pattern; the anthesis pedicel AZs specifically express *BLIND* (*Bl*), *GOBLET* (*GOB*), *Ls* and a tomato *WUSCHEL* homologue (*LeWUS*), and this expression is not present in the pedicels of AZ-lacking mutants [10]. The function of these transcription factor genes in pedicel AZs is still unknown, but these genes are well known to play crucial roles in shoot apical or axillary meristems [10,12,30-32]. Their AZ-specific expression suggests that these transcription factors may act at the pre-abscission step, possibly to prevent flower abscission or to establish competence to respond to abscission signaling.

**Figure 1:**
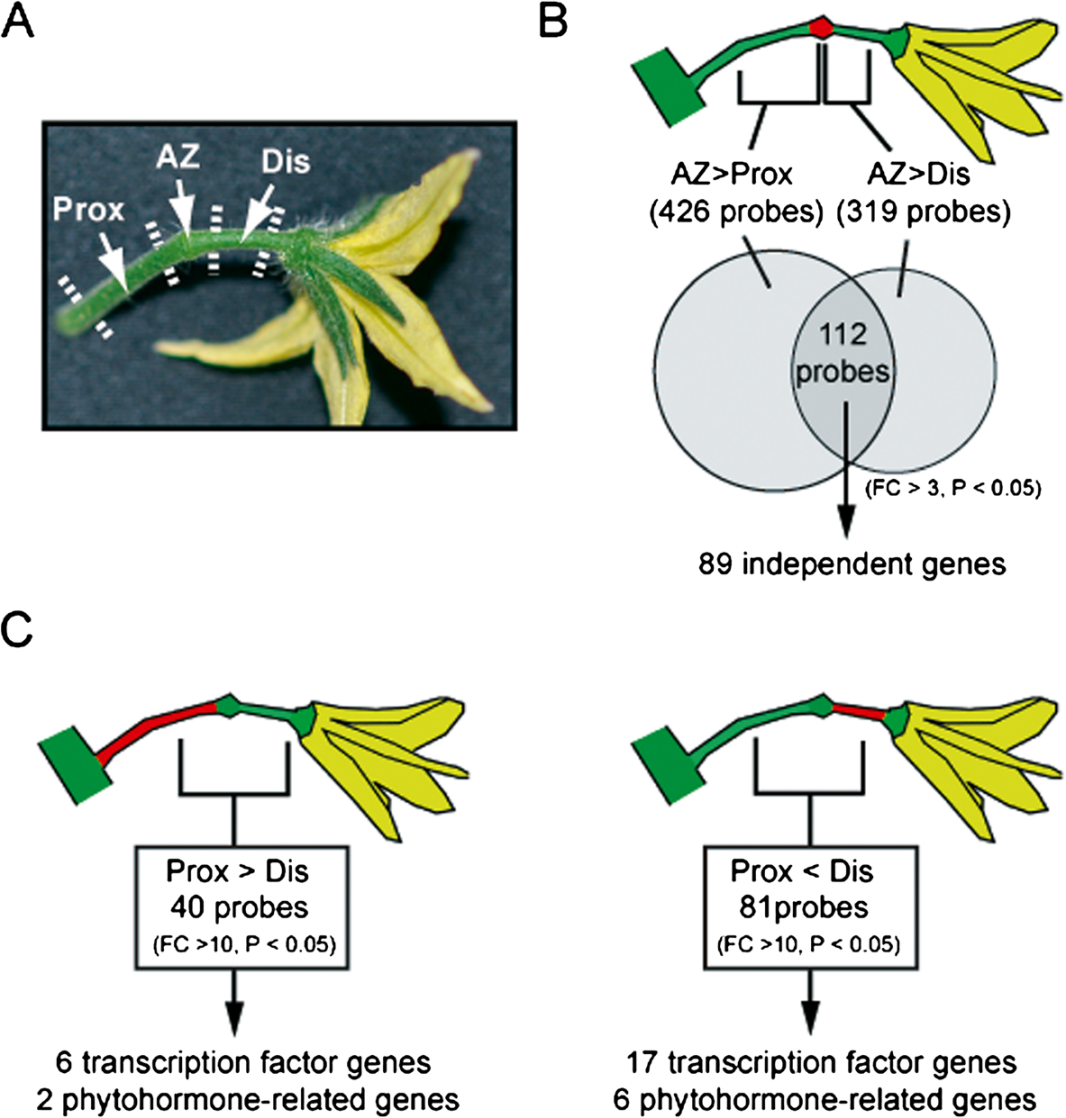
**Screening for genes differentially expressed in different tomato pedicel regions by expression microarray analysis.** (**A**) Flower pedicel regions used in this study. Tomato abscission zones (AZs) form in an intermediate region in the pedicels and have a knuckle-like structure in which a groove forms for abscission. The pedicel region between the AZ and the main stem of the inflorescence is referred to as the proximal region (Prox) and the region between the AZ and the flower is referred to as the distal region (Dis). RNA from each region was extracted from five to twenty pedicels at anthesis. (**B**) A schematic of the genome-wide transcriptome screen for genes up-regulated in the AZ. Expression was compared between AZ and Prox or Dis at anthesis by microarray analyses with 3 independently prepared samples. Two circles in the Venn diagram indicate the number of probes showing higher signal from AZ than that from Prox or Dis, and the 112 probes in the overlap were further investigated in this study. By merging results for probes encoding the same gene, we found 89 independent genes up-regulated in the AZ. (**C**) A schematic of the screening for transcription factor and phytohormone-related genes differentially expressed between Prox and Dis. The expression profiles of Prox and Dis examined by microarray analyses were compared and genes for transcription factors or phytohormone-related activity were selected from the genes exhibiting significantly different expression patterns between the two regions.

To identify additional genes potentially involved in the abscission of tomato pedicels at anthesis, here we performed transcriptome analyses comparing the expression profiles in AZs with two flanking pedicel regions, on the flower side (distal region; Dis) and on the inflorescence side (proximal region; Prox, Figure 1A). Examination of the specific expression properties of these pedicel regions provided insights on the properties of each pedicel region and on cell activity in the pre-abscission state. In addition, we further characterized four previously identified transcription factors, examining the detailed expression patterns of *LeWUS, Bl, GOB* and *Ls* at anthesis. Our results indicated that these four genes showed significant expression changes when abscission was induced, suggesting that these genes play pivotal roles in the onset of abscission.

## Results

### Comparative transcriptome analysis revealed genes up-regulated in the AZ of tomato flower pedicels at anthesis

AZ cells of a tomato flower are recognized even at the early stage of flower primordium development; at the flower anthesis stage, six to eight layers of cells are observed in the AZ and the flower pedicels have acquired the competence to respond to abscission-promoting signals [27,33,34]. To identify genes preferentially expressed in the AZ of tomato flower pedicels at anthesis, we used the Agilent tomato 44K oligonucleotide DNA microarray to perform transcriptome assays comparing AZs and the non-AZ pedicel regions, Dis and Prox (Figure 1A). Microarray experiments were performed on three independently prepared samples and probes showing at least a 3-fold change (*p*<0.05) in signal intensity were selected. As a result, we found that 426 probes showed higher signal intensity in the AZ than in Prox, and 319 probes were higher in the AZ than in Dis (Additional file 1 and 2). In total, 112 probes showed higher signal intensity in the AZ than in both Prox and Dis (Figure 1B). Of the EST sequences for the 112 probes, 105 ESTs represented 82 International Tomato Annotation Group (ITAG2)-predicted genes, but the remaining 7 ESTs have not yet been assigned to any predicted genes (Table 1). Here we regarded the 82 predicted genes and 7 non-assigned ESTs as independent genes, and further examined the functions of these 89 genes. To verify the microarray assay results, we arbitrarily selected 10 genes out of the 89 genes and analyzed expression of the 10 genes by reverse transcription PCR (RT-PCR) (Additional file 3). The expression specificities within the three pedicel regions showed good consistency between the RT-PCR and the microarray results.

**Table 1:** Genes up-regulated in the tomato pedicel abscission zone (AZ) compared with the proximal (Prox) and distal (Dis) regions of the pedicel

					**AZ > Prox**		**AZ > Dis**	
**Tomato Gene ID**	**Probe ID**	**Gene name / EST accession number**	**Arabidopsis homologue**	**Annotation**	**Fold change (log**_**2**_**)**	***p*-value**	**Fold change (log**_**2**_**)**	***p*-value**
***Transcription factor***								
Solyc02g083950	A_96_P013906	LeWUS AJ538329	AT2G17950	WUS (WUSCHEL)	8.5	0.002	8.3	0.002
Solyc03g117130^a^	A_96_P240288	SlERF52 AK327476	AT5G25190	ethylene-responsive element-binding protein, putative	7.3	< 0.001	6.7	0.002
Solyc07g066250	A_96_P000266	Ls AF098674	AT1G55580	LAS (Lateral Suppressor)	6.0	0.008	5.9	0.008
Solyc11g069030	A_96_P012746	Bl AF426174	AT5G57620	MYB36 (myb domain protein 36)	5.5	0.008	4.5	0.007
Solyc07g062840	A_96_P226779	GOB FJ435163	AT5G53950	ANAC098 | CUC2 (CUP-SHAPED COTYLEDON 2)	5.2	0.002	3.8	0.003
Solyc09g066360	A_96_P115892	SlERF56 TA54084_4081	AT3G23240	ERF1 (ETHYLENE RESPONSE FACTOR 1)	4.9	0.014	2.3	0.019
Solyc01g080960^a^	A_96_P017531	AK330067	AT4G17800	DNA-binding protein-related | Predicted AT-hook DNA-binding family protein	4.7	0.014	4.1	0.009
Solyc08g076010	A_96_P172039	BE431711	AT5G45580	transcription factor | Homeodomain-like superfamily protein	3.6	0.017	3.1	0.011
Solyc02g085500^a^	A_96_P013166	OVATE AK247861	AT2G18500	ATOFP7 (ARABIDOPSIS THALIANA OVATE FAMILY PROTEIN 7)	2.8	0.002	1.8	< 0.001
Solyc01g102980	A_96_P226304	CK715116	AT1G75240	AtHB33 (ARABIDOPSIS THALIANA HOMEOBOX PROTEIN 33)	2.3	0.022	1.7	0.022
Solyc08g065420	A_96_P014181	BL4 AF375967	AT4G32980	ATH1 (ARABIDOPSIS THALIANA HOMEOBOX GENE 1)	2.1	0.019	4.0	0.018
Solyc08g078180	A_96_P030681	SlERF68 AW034080	AT5G47220	ERF2 (ETHYLENE RESPONSIVE ELEMENT BINDING FACTOR 2)	2.0	0.010	3.2	0.044
Solyc05g005090	A_96_P012291	TKn3 U76408	AT1G23380	KNAT6 (KNOTTED1-LIKE HOMEOBOX GENE 6)	1.9	0.018	4.1	< 0.001
***Phytohormone metabolism/signaling/response***								
Solyc07g063850	A_96_P127252	AK319847	AT5G54510	GH3.6, DFL1 | DFL1 (DWARF IN LIGHT 1); indole-3-acetic acid amido synthetase	3.7	0.003	1.6	0.028
Solyc01g109150	A_96_P011856	AF461042	AT5G42650	AOS (ALLENE OXIDE SYNTHASE)	3.1	0.039	3.6	0.004
Solyc03g070380^a^	A_96_P257477	AK328818	AT2G23620	MES1 (METHYL ESTERASE 1); hydrolase, acting on ester bonds / methyl indole-3-acetate esterase/ methyl jasmonate esterase/ methyl salicylate esterase	2.5	< 0.001	1.6	0.004
Solyc03g120060	A_96_P054606	AB223041	AT2G26710	BAS1 (PHYB ACTIVATION TAGGED SUPPRESSOR 1)	3.1	0.011	3.2	0.015
Solyc02g089160	A_96_P173339	Dwarf BE433666	AT3G30180	BR6OX2 (BRASSINOSTEROID-6-OXIDASE 2)	3.3	0.024	2.8	0.048
Solyc12g042500	A_96_P137617	AI779761	AT5G59845	gibberellin-regulated family protein	2.0	0.020	1.7	0.002
***Cell wall hydrolysis/modification***								
Solyc12g096750	A_96_P012556	TAPG4 U70481	AT3G59850	polygalacturonase, putative / pectinase, putative	8.7	0.016	8.6	0.022
Solyc12g019180	A_96_P128437	NP000616	AT3G59850	polygalacturonase, putative / pectinase, putative	3.0	0.005	2.1	0.017
Solyc01g094970	A_96_P232064	AK323960	AT3G61490	glycoside hydrolase family 28 protein / polygalacturonase (pectinase) family protein	4.5	0.012	4.4	0.015
Solyc03g093390	A_96_P085969	EXPB2 DQ205653	AT1G65680	ATEXPB2 (ARABIDOPSIS THALIANA EXPANSIN B2)	3.7	0.040	2.9	0.047
Solyc03g006700	A_96_P077754	TA38392_4081	AT5G05340	peroxidase, putative	2.6	< 0.001	4.3	0.004
***Defense function***								
Solyc10g017980	A_96_P216559	TA36496_4081	AT3G12500	ATHCHIB (ARABIDOPSIS THALIANA BASIC CHITINASE)	4.2	0.004	6.7	< 0.001
Solyc07g009530	A_96_P035581	BW687719	AT3G12500	ATHCHIB (ARABIDOPSIS THALIANA BASIC CHITINASE)	3.5	0.014	3.3	0.034
Solyc10g055800	A_96_P198339	BI209334	AT3G12500	ATHCHIB (ARABIDOPSIS THALIANA BASIC CHITINASE)	1.8	0.004	2.5	0.017
Solyc08g080640	A_96_P089294	TA36568_4081	AT4G11650	ATOSM34 (osmotin 34)	5.2	0.037	2.2	< 0.001
Solyc08g080650	A_96_P156561	pr p23 AK322366	AT4G11650	ATOSM34 (osmotin 34)	3.6	0.048	3.3	0.011
Solyc01g106620	A_96_P076884	AK324158	AT2G14580	ATPRB1 (ARABIDOPSIS THALIANA BASIC PATHOGENESIS-RELATED PROTEIN 1)	5.4	0.036	6.0	0.011
Solyc09g090970	A_96_P077909	AK326776	AT1G24020	MLP423 (MLP-LIKE PROTEIN 423)	3.4	0.046	2.2	0.033
Solyc01g097270	A_96_P148266	AW037799	AT3G04720	PR4 (PATHOGENESIS-RELATED 4)	2.6	0.020	1.7	0.028
***Lipid metabolism***								
Solyc01g090350	A_96_P084944	BW688588	AT4G33355	lipid binding	2.3	0.002	1.8	0.004
Solyc10g085740^a^	A_96_P204184	BI928574	AT5G03820	GDSL-motif lipase/hydrolase family protein	4.2	0.041	5.3	0.035
Solyc12g044950	A_96_P191076	cevi19 AK323674	AT3G12120	FAD2 (FATTY ACID DESATURASE 2)	3.5	0.046	3.3	0.042
Solyc02g086490	A_96_P038006	GO374663	AT3G01570	glycine-rich protein / oleosin	3.4	0.018	3.6	0.016
Solyc03g083990	A_96_P077599	AI777049	AT2G45180	protease inhibitor/seed storage/lipid transfer protein (LTP) family protein	3.2	0.010	3.7	0.029
Solyc09g065240	A_96_P144011	AW030712	AT3G63200	PLP9 (PATATIN-LIKE PROTEIN 9)	1.6	0.031	2.7	0.005
***Transporter/Channel***								
Solyc02g085170	A_96_P127407	TA56865_4081	AT1G19450	integral membrane protein, putative / sugar transporter family protein	3.8	0.014	4.1	0.016
Solyc01g103030	A_96_P114407	AK325211	AT1G59740	proton-dependent oligopeptide transport (POT) family protein	1.9	0.023	2.0	0.034
Solyc10g084950^a^	A_96_P231269	DB697130	AT2G37900	proton-dependent oligopeptide transport (POT) family protein	2.6	0.005	2.5	0.026
Solyc07g063930	A_96_P137262	AI778966	AT3G20660	AtOCT4 (Arabidopsis thaliana ORGANIC CATION/CARNITINE TRANSPORTER4)	3.0	0.002	1.7	0.003
Solyc03g005980	A_96_P190219	BG134199	AT4G18910	NIP1;2 (NOD26-LIKE INTRINSIC PROTEIN 1;2); arsenite transmembrane transporter/ water channel	1.8	0.006	2.4	0.016
Solyc01g010080	A_96_P054371	AW218955	AT4G32650	ATKC1 (ARABIDOPSIS THALIANA K+ RECTIFYING CHANNEL 1)	3.5	0.018	2.8	0.027
***Others***								
Solyc12g013820^a^	A_96_P147601	AK325708	AT1G53020	UBC26 (UBIQUITIN-CONJUGATING ENZYME 26)	2.2	< 0.001	2.9	0.017
Solyc03g034020	A_96_P202249	BI925250	AT4G36550	binding / ubiquitin-protein ligase	2.1	0.014	1.6	0.009
Solyc01g010250	A_96_P215689	BP892102	AT1G13700	glucosamine/galactosamine-6-phosphate isomerase family protein	2.1	0.006	1.7	0.002
Solyc12g042470	A_96_P109252	TA51045_4081	AT1G59950	aldo/keto reductase, putative	4.2	0.018	3.8	0.045
Solyc00g071180	A_96_P016051	AF083253	AT3G12490	ATCYSB | cysteine protease inhibitor, putative / cystatin, putative	1.7	< 0.001	1.6	0.002
Solyc09g089500	A_96_P141479	AJ319916	AT2G38870	protease inhibitor, putative	1.8	0.044	2.7	0.035
Solyc01g087820	A_96_P139602	AK324419	AT5G67090	subtilase family protein	2.0	0.005	2.2	0.027
Solyc03g112420	A_96_P203679	BI927360	AT1G64310	pentatricopeptide (PPR) repeat-containing protein	2.2	0.023	1.8	0.017
Solyc02g086270	A_96_P238007	DB715353	AT1G67025	unknown	1.8	0.010	1.7	0.007
Solyc01g109720	A_96_P043946	AK323257	AT2G18360	hydrolase, alpha/beta fold family protein	2.8	0.006	2.5	0.003
Solyc09g008740	A_96_P160421	AW625490	AT2G22880	VQ motif-containing protein	2.8	0.012	2.1	0.022
Solyc12g005700	A_96_P016566	BT012940	AT2G32280	unknown protein	2.4	0.011	5.4	0.004
Solyc10g007310	A_96_P084909	BW687670	AT2G42610	LSH10 (LIGHT SENSITIVE HYPOCOTYLS 10)	2.3	0.017	3.6	0.002
Solyc03g114130	A_96_P219824	AK329003	AT3G01430	unknown	1.8	0.002	1.9	0.003
Solyc01g081270	A_96_P152326	AK320517	AT3G09270	ATGSTU8 (GLUTATHIONE S-TRANSFERASE TAU 8)	1.6	0.011	1.6	0.041
Solyc08g059710	A_96_P039956	AK246959	AT3G11760	unknown protein	2.8	0.011	2.4	0.014
Solyc06g062800	A_96_P214714	BP889760	AT3G13920	EIF4A1 (EUKARYOTIC TRANSLATION INITIATION FACTOR 4A1)	2.2	0.015	2.8	0.023
Solyc12g097060	A_96_P065581	BI210672	AT3G23930	unknown protein	2.4	0.013	4.0	0.018
Solyc04g074300	A_96_P191679	BG626643	AT3G57490	40S ribosomal protein S2 (RPS2D)	3.3	0.018	3.3	< 0.001
Solyc08g023270	A_96_P259047	TC213163	AT4G03620	myosin heavy chain-related	1.8	0.016	2.9	0.047
Solyc10g054440^a^	A_96_P089580	ADC1 AK319876	AT4G34710	ADC2 (ARGININE DECARBOXYLASE 2)	1.9	< 0.001	2.0	0.010
Solyc12g010960	A_96_P127072	TA56779_4081	AT5G16990	NADP-dependent oxidoreductase, putative	2.7	0.007	1.7	0.039
Solyc06g082030	A_96_P226684	CK720539	AT5G58660	oxidoreductase, 2OG-Fe(II) oxygenase family protein	4.7	0.006	2.7	0.004
Solyc03g114820	A_96_P112182	TA53188_4081	AT5G17390	universal stress protein (USP) family protein	2.1	0.015	1.8	< 0.001
Solyc04g050790	A_96_P170549	BE344440	AT5G18310	unknown protein	1.9	0.005	2.9	0.003
Solyc01g095960	A_96_P129692	AI483484	AT5G53390	unknown protein	3.9	0.022	1.8	0.022
Solyc06g075690	A_96_P235420	AK326143	AT5G59790	unknown protein	2.4	0.049	1.6	0.036
Solyc09g055950	A_96_P108797	BI211136	ATCG00270	PSBD | PSII D2 (PHOTOSYSTEM II REACTION CENTER PROTEIN D2)	4.1	0.019	3.9	0.038
Solyc09g091400^a^	A_96_P196349	BI204004	AT3G24240	leucine-rich repeat transmembrane protein kinase, putative	4.0	0.020	3.5	0.039
Solyc02g084670	A_96_P112502	TA53264_4081	no hits found		5.7	0.018	4.8	< 0.001
Solyc01g091400^a^	A_96_P197999	BI208492	no hits found		5.4	0.003	5.2	0.011
Solyc02g031990	A_96_P224639	CD002083	no hits found		4.0	0.013	3.0	0.038
Solyc03g070430	A_96_P106729	AK247727	no hits found		3.7	0.020	3.8	0.005
ND	A_96_P181929	BF097523	no hits found		3.3	0.007	3.3	0.001
Solyc03g034360	A_96_P110867	TA52360_4081	no hits found		3.0	0.043	3.1	0.031
Solyc08g074680	A_96_P207909	BM410550	no hits found		2.8	0.005	2.9	0.012
Solyc04g055050	A_96_P062011	AW222670	no hits found		2.8	0.001	1.9	0.001
ND	A_96_P261677	TC215751	no hits found		2.3	0.022	2.2	0.033
ND	A_96_P246260	AK325898	no hits found		2.1	0.028	1.6	0.012
ND	A_96_P104119	AK325900	no hits found		2.0	0.016	1.9	0.021
ND	A_96_P204674	BI929508	no hits found		2.0	0.047	3.5	0.030
Solyc07g056280	A_96_P131347	AI487047	no hits found		1.8	0.008	2.1	0.015
ND	A_96_P069949	DB717716	no hits found		1.6	0.026	3.6	0.025
Solyc03g113910	A_96_P201699	BI924325	no hits found		1.6	0.049	3.6	0.026
ND	A_96_P160681	AW626075	no hits found		1.6	0.013	2.0	0.011

If more than two probes derived from the same gene were selected, only the result of the probe indicating the highest fold change value was shown in this table. Each assay result is shown in Additional file 1 and 2. Genes examined by RT-PCR are marked with lower case “a”.

Based on the annotation of their Arabidopsis homologues, we inferred the function of the 89 genes preferentially expressed in the AZ. From the putative functions of these 89 genes, we found 6 major functional groups: transcription factors (13 genes), phytohormone metabolism, signaling and response (6 genes), cell wall-degrading or modification (5 genes), defense function (8 genes), lipid metabolism (6 genes) and transporter/channel proteins (6 genes) (Table 1).

We also identified genes for which expression in the AZ was lower than that in Prox and Dis. The genes showing AZ expression levels of less than one-third of that detected in both Prox and Dis (p<0.05) were selected. This group included only four ESTs, BF114405, TA41413_4081, AK248036, and AK324977 (Additional file 4). AK324977 encodes a homologue of a putative auxin-responsive protein, whereas the others showed no sequence similarity to any known genes. The result was insufficient to assess specific features of the AZ cells thus we did not analyze these four further.

### Transcription factor genes specifically up-regulated in the AZ

We previously reported that pedicel AZ cells at anthesis express the transcription factor genes *Bl, GOB, Ls* and *LeWUS*, which have been identified as regulators of apical or axillary meristem development. In addition to these 4 genes, here we found 9 other transcription factor genes that were preferentially expressed in pedicel AZs at anthesis (Table 1). The 9 identified genes belong to 7 transcription factor families: a KNOX family gene (*Tomato Knotted 3* [*TKn3*]), a BELL family gene (*bell-like homeodomain protein 4* [*BL4*]), a zinc finger-homeodomain (ZF-HD) family gene (CK715116), an OVATE family gene (*OVATE*), 3 ethylene responsive transcription factor (ERF) family genes (*SlERF52*, *SlERF56* and *SlERF68*), an AT-hook family gene (AK330067) and a homeodomain-like superfamily gene (BE431711) (Table 1). *OVATE*, *SlERF52* and CK715116 are expressed in an *MC* and *JOINTLESS* dependent manner in tomato pedicels, similar to *Bl, GOB, Ls* and *LeWUS*[10]*.* Previous studies showed that several transcription factor families described here play roles in organ abscission. For example, the KNOX family transcription factors, which were first identified to regulate shoot meristem identity [35], were also shown to be involved in abscission processes in floral organs [36,37]. Also, the BELL family gene *qSH1* regulates formation of the abscission zone in rice seed shattering [19]. Some of the transcription factor families found in AZs are involved in phytohormone metabolism and signaling pathways. For example, the CK715116 encoding protein showed sequence similarity to the Arabidopsis ZF-HD family gene *HOMEOBOX PROTEIN33*, which functions in the abscisic acid (ABA) response pathway [38]. The AT-hook family controls inflorescence formation, senescence, and gibberellin (GA) metabolism [39-41]. Members of the ERF family were originally identified as regulators of ethylene signaling but are now known to respond to abiotic and biotic stresses and regulate lipid metabolism and development [42-45]. The OVATE family proteins have been identified as regulators of cell elongation, and tomato *OVATE* regulates fruit shape [46,47].

### Phytohormone related genes specifically up-regulated in the AZ

Because pedicel abscission at the pre-abscission stage is prevented by auxin, we expected that genes related to auxin activities would be expressed predominantly in the AZ. Indeed, AK328818 and AK319847, which are homologues of *METHYLESTERASE1* (*MES1*) and *DWARF IN LIGHT1* (*DFL1*)/ *auxin-inducible Gretchen Hagen 3.6* (*GH3.6*), respectively, showed higher transcript levels in the AZ than in Prox and Dis (Table 1). *MES1* is implicated in conversion of a storage form of indole-3-acetic acid (IAA) into the active form and *DFL1* encodes an IAA amido synthetase, which produces a storage form of IAA from the active form [48-50]. In addition, several genes involved in pathways related to diverse phytohormones showed higher expression in the AZ than in non-AZ tissues; these include genes involved in jasmonate (JA) metabolism (AF461042), brassinosteroid metabolism and biosynthesis (*Dwarf* and AB223041) and GA signaling regulation (AI779761).

### Cell wall degrading and remodeling genes specifically up-regulated in the AZ

**Figure 2:**
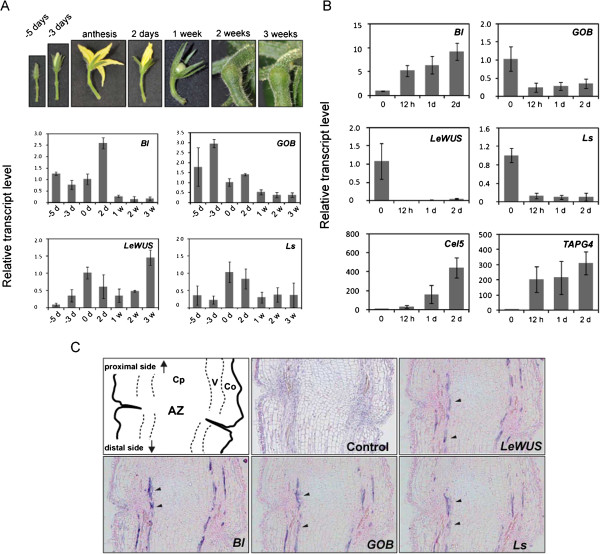
**Expression of *Bl*, *GOB*, *LeWUS *and *Ls *in tomato pedicels.** (**A**) Expression of *Bl*, *GOB*, *LeWUS* and *Ls* during pedicel development. Expression of the genes in pedicels at 5 and 3 days before anthesis, at anthesis and at 2 days to 3 weeks after anthesis was examined by qRT-PCR. Levels of transcripts of each gene are shown as fold-change values compared to the sample harvested at anthesis. (**B**) Expression of *Bl*, *GOB*, *LeWUS* and *Ls* in response to an abscission stimulus. Anthesis flowers were removed from the pedicels to induce abscission and then the pedicel AZs were harvested at 0 hours, 12 hours, 1 day and 2 days after flower removal. The expression of *Bl*, *GOB*, *LeWUS* and *Ls* was examined by qRT-PCR. Error bars indicate standard deviation of biological triplicates. At 1 day after flower removal, approximately 10% of pedicels were abscised and then the rate increased to 36% at 2 days after (Additional file 8). Both attached and detached pedicels were examined simultaneously. As positive controls, expression analyses were performed for *Cel5* and *TAPG4*, which are up-regulated after flower removal [27]. Levels of transcripts of each gene are shown as fold-change values compared to the 0 hour sample. Error bars indicate standard deviation of biological triplicates. (**C**) Expression of *Bl*, *GOB*, *Ls*, and *LeWUS* in a flower pedicel AZ at anthesis. Dig-labeled antisense probes were hybridized to serial sections of a pedicel AZ at anthesis. Transcripts of these genes, indicated by arrowheads, were found in several lines of cells within the vascular bundles. The upper left panel is a schematic of an AZ. The control section was stained with a hematoxylin and eosin stain solution. AZ; abscission zone, Co; Cortex, V; Vascular bundle, and Cp; Central parenchyma.

Our transcriptome analyses showed that several genes for cell wall degrading and remodeling factors were expressed at higher levels in the AZ than in the non-AZ tissues. These include genes for polygalacturonase (PG), peroxidase and expansin. PG, an enzyme that hydrolyzes cell wall pectin, has been demonstrated to promote abscission in various plant organs [51,52] and PG genes are strongly up-regulated at the onset of abscission in tomato pedicels [27] and citrus (*Citrus clementina*) leaves [29]. In tomato pedicels, PG expression is strictly limited to the AZ after initiation of abscission [53]. Expansin is a cell wall remodeling protein and reportedly regulates abscission of leaflets in elderberry (*Sambucus nigra*) and pedicels in Arabidopsis [25,26]. Gene expression or enzyme activity of peroxidases is detected during abscission in tobacco (*Nicotiana tabacum*) flower pedicels and citrus leaves [29,54]. Although the expression of the genes for PG, expansin and peroxidase were expressed preferentially in AZs, the expression levels of the genes at the pre-abscission stage was much lower than after the onset of abscission (Figure 2B) [25,27]. Thus, these proteins may have different functions between pre-abscission and during abscission. During the pre-abscission stage, these genes may be involved in AZ tissue maintenance or thickening of the AZ by remodeling cell wall components. It is also possible that the PG activity at anthesis may be too low to have any significant effect on cells, because the expression level was much lower than that during the abscission activated stage (Figure 2B).

### Defense-related and lipid metabolism genes specifically up-regulated in the AZ

Several *pathogenesis-related* (*PR*) genes are reportedly expressed at the site where organs will be shed during abscission [1]. The *PR* genes are expected to function in defense to prevent potential pathogen infections during abscission [1]. Many *PR* genes are also expressed in unstressed tissues such as poplar leaves and tobacco flowers, possibly for development or basal defense activity against opportunistic pathogen invasion [55,56]. Our transcriptome analysis also revealed that homologues of defense-related genes encoding basic chitinases (TA36496_4081, BW687719 and BI209334), PR4 (AW037799), basic PR1 (AK324158), osmotin proteins (TA36568_4081 and pr p23) and a major latex protein*–*like protein (AK326776) were preferentially expressed in AZs at anthesis.

Lipids may act as an additional defense against pathogen infection or dehydration at abscission. An anatomical study revealed that the clefts in pedicel AZs are filled with lipid compounds such as cutin [57] and our previous study showed that many lipid metabolism genes were down-regulated in the pedicels of non-AZ-forming plants (antisense-*MC* transgenic plants and *jointless* mutants) compared with wild-type plants [10]. Accordingly, we found that 6 homologues associated with lipid metabolism were preferentially expressed in the AZ; the genes encode a lipid binding protein (BW688588), a GDSL-motif lipase/hydrolase (BI928574), a fatty acid desaturase (*citrus exocortis viroid-inducible19* [*cevi19*]), a protease inhibitor/seed storage/lipid transfer protein (AI777049), a glycine rich protein/oleosin (GO374663), and a patatin-like protein (AW030712) (Table 1).

### Genes differentially expressed between Prox and Dis

Previous investigations showed that, after the onset of abscission, the Prox and Dis tissues show distinct expression patterns for several abscission-related genes such as *PG*, *β-1,4-glucanase* (*cellulose*) and *ribonuclease* at anthesis [51,58,59]. In this study, we analyzed expression pattern differences between the two tissues at the anthesis stage, or pre-abscission stage. By using fold-change > 3 (*p*<0.05) as a cutoff, we found that 629 probes showed higher signal intensity in Prox than in Dis (Figure 1C, Additional file 5) and 392 probes were higher in Dis than in Prox (Figure 1C, Additional file 6). Subsequently, among these genes, we focused on transcription factors and phytohormone-related genes. By using more strict criteria, fold-change > 10 (*p*<0.05), as a cutoff, we identified 6 transcription factor genes and 2 phytohormone-related genes that were preferentially expressed in Prox (Table 2), and 17 transcription factor genes and 6 phytohormone-related genes that were preferentially expressed in Dis (Table 3). We also used RT-PCR to verify the results of the microarray analyses for 11 arbitrarily selected genes and found that the RT-PCR results were consistent with the microarray data (Additional file 7).

**Table 2:** Genes preferentially expressed in a tomato pedicel proximal region (Prox) compared with the distal region (Dis)

					**Prox > Dis**		**Prox > AZ**	
**Tomato gene ID**	**Probe ID**	**Gene name / EST accession number**	**Arabidopsis homologue**	**Annotation**	**Fold change (log**_**2**_**)**	***p*-value**	**Fold change (log**_**2**_**)**	***p*-value**
***Transcription factor***								
Solyc12g009580^a^	A_96_P138787	AI782101	AT2G26580	YAB5 (YABBY5)	5.0	0.015	3.2	0.074
Solyc01g096070	A_96_P108377	TA50096_4081	AT4G23980	ARF9 (AUXIN RESPONSE FACTOR 9)	3.4	0.005	0.3	0.059
Solyc08g076820	A_96_P101994	AK323669	AT5G46690	bHLH071 (basic-helix-loop-helixHLH protein 71)	3.4	0.002	1.5	0.003
Solyc06g005310	A_96_P124087	TA56053_4081	AT3G46130	MYB111 (MYB DOMAIN PROTEIN 111)	3.6	0.036	2.5	0.007
Solyc12g056460^a^	A_96_P182539	SlMBP14 BF098196	AT2G45660	SOC1 | AGL20 (AGAMOUS-LIKE 20)	3.7	0.018	2.0	0.069
Solyc06g065820^a^	A_96_P012741	SlERF1 AY077626	AT5G25190	ethylene-responsive element-binding protein, putative	3.7	0.006	1.4	0.007
***Phytohormone-related function***								
Solyc08g014000^a^	A_96_P012631	LOXA U09026	AT1G55020	LOX1	3.6	0.008	−0.8	0.365
Solyc11g011210	A_96_P190874	RSI-1 AK324086	AT3G02885	GASA5 (GAST1 PROTEIN HOMOLOG 5)	4.2	0.019	−0.7	0.014

If more than two probes derived from the same gene were selected, only the result of the probe indicating the highest fold change value was shown in this table. Each assay result is shown in Additional file 5. Genes examined by RT-PCR are marked with lower case “a”.

**Table 3:** Genes up-regulated in the tomato pedicel distal region (Dis) compared with the proximal region (Prox)

					**Dis > Prox**		**Dis > AZ**	
**Tomato gene ID**	**Probe ID**	**Gene name / EST accession number**	**Arabidopsis homologue**	**Annotation**	**Fold change (log**_**2**_**)**	***p*-value**	**Fold change (log**_**2**_**)**	***p*-value**
***Transcription factor***								
Solyc02g089200^a^	A_96_P195359	TM29 BG734619	AT5G15800	SEP1 (SEPALLATA1)	8.8	0.004	2.4	< 0.001
Solyc05g015750^a^	A_96_P000181	TDR5 AY294330	AT1G24260	SEP3 (SEPALLATA3)	7.4	0.018	4.6	< 0.001
Solyc07g006880	A_96_P040576	AK326074	AT5G57520	ZFP2 (ZINC FINGER PROTEIN 2)	7.0	0.006	3.0	<0.001
Solyc01g093960^a^	A_96_P204169	BI203609	AT2G45650	AGL6 (AGAMOUS-LIKE 6)	6.5	0.005	0.4	0.041
Solyc06g073920^a^	A_96_P249912	AK328263	AT1G08465	YAB2 (YABBY2)	5.6	< 0.001	1.2	0.013
Solyc02g085630	A_96_P039876	AI898032	AT4G36740	ATHB40 (ARABIDOPSIS THALIANA HOMEOBOX PROTEIN 40)	5.5	0.023	0.6	0.059
Solyc02g086690	A_96_P163816	AW737355	AT5G40350	MYB24 (myb domain protein 24)	5.1	< 0.001	1.5	0.009
Solyc12g038510^a^	A_96_P114197	SlMBP21 TA53678_4081	AT5G15800	SEP1 (SEPALLATA1)	5.0	0.001	0.2	0.051
Solyc12g100150	A_96_P191189	AK328730	AT1G31320	LBD4 (LOB DOMAIN-CONTAINING PROTEIN 4)	4.8	0.001	1.1	0.028
Solyc01g106250	A_96_P113847	TA53587_4081	AT5G60142	DNA binding	4.6	0.010	1.2	0.024
Solyc02g085910	A_96_P050501	AK328874	AT3G02550	LBD41 (LOB DOMAIN-CONTAINING PROTEIN 41)	4.3	0.006	0.8	0.127
Solyc04g079360	A_96_P185909	BG126724	AT3G50060	MYB77 (myb domain protein 77)	4.2	0.008	1.8	0.005
Solyc04g081000^a^	A_96_P206659	TAP3 DQ674532	AT3G54340	AP3 (APETALA 3)	4.1	0.004	1.2	0.017
Solyc07g008020	A_96_P212749	AK319758	AT4G32280	IAA29 (INDOLE-3-ACETIC ACID INDUCIBLE 29)	4.0	0.005	1.8	0.076
Solyc03g044300	A_96_P172149	SlAP2a AK326004	AT4G36920	AP2 (APETALA 2)	3.8	0.007	2.0	0.022
Solyc07g066330	A_96_P252807	TC202847	AT1G56010	ANAC022 | NAC1	3.4	0.012	0.1	0.494
Solyc07g063410	A_96_P046081	AK323372	AT4G27410	ANAC072 | RD26 (RESPONSIVE TO DESICCATION 26)	3.3	0.018	0.9	0.101
***Phytohormone-related function***								
Solyc02g064690^a^	A_96_P209009	BP875651	AT4G37580	HLS1 (HOOKLESS 1)	4.8	0.003	2.4	0.001
Solyc02g092490	A_96_P079464	AK247718	AT4G37580	HLS1 (HOOKLESS 1)	3.6	0.019	1.2	0.101
Solyc01g107400	A_96_P042196	BW692346	AT2G14960	GH3.1	4.0	0.035	1.7	0.008
Solyc07g026650	A_96_P020931	SlACO5 AJ715790	AT2G19590	ACO1 (ACC OXIDASE 1)	4.4	0.020	0.2	0.553
Solyc10g017990	A_96_P028296	BG130984	AT5G56970	CKX3 (CYTOKININ OXIDASE 3)	4.3	0.026	−1.5	0.291
Solyc12g008900	A_96_P107909	TA49618_4081	AT5G56970	CKX3 (CYTOKININ OXIDASE 3)	4.3	0.017	−1.5	0.296

If more than two probes derived from the same gene were selected, only the result of the probe indicating the highest fold change value was shown in this table. Each assay result is shown in Additional file 6. Genes examined by RT-PCR are marked with lower case “a”.

### Transcription factor genes differentially expressed between Prox and Dis

Among the genes preferentially expressed in Dis, we found five MADS-box genes (BI203609, *MADS-box 5* [*TDR5 or TM5*], *TM29*, *MADS-box Protein 21* [*SlMBP21*], and *Tomato APETALA3* [*TAP3*]), which are homologous to *AGAMOUS-LIKE6* (*AGL6*), *SEPALATA1* (*SEP1*), *SEPALATA3* (*SEP3*) or *AP3*, the Arabidopsis regulators of flower organ identity (Table 3). An *APETALA2* (*AP2*) homologue (*SlAP2a*) was also found among the genes preferentially expressed in Dis. Arabidopsis *AP2* encodes a transcription factor belonging to the AP2 family and regulates flower organ identity [60]; the tomato homologue *SlAP2a* has been shown to be a negative regulator of ethylene biosynthesis during fruit ripening [61]. Recently, a rice *AP2* homologue has been shown to regulate AZ development for seed shattering [18]. By contrast, among the transcription factor genes preferentially expressed in Prox, we found a homologue of Arabidopsis *SUPPRESSOR OF OVEREXPRESSION OF CONSTANS 1* (*SOC1*), which encodes a MADS-box protein regulating floral meristem identity (*SlMBP14*; Table 2) [62,63].

Several transcription factor genes implicated in auxin-regulated signaling pathways showed differential expression between Prox and Dis. For example, an auxin response factor (ARF) family gene (TA50096_4081) was preferentially expressed in Prox. The genes preferentially expressed in Dis include homologues that encode members of the auxin/indole-3-acetic acid (AUX/IAA) family (AK319758), NAC family (TC202847), and MYB family (BG126724). ARF family proteins are implicated as regulators of auxin signaling [64] and Arabidopsis *ARF1*, and *ARF2* are redundantly required to promote floral organ abscission [65]. AK319758 encodes a homologue of Arabidopsis IAA29, which is involved in auxin-mediated elongation of hypocotyls [66]. BG126724 is a *MYB77* homologue that modulates auxin signaling [67]. TC202847 shows similarity to *NAC1*, which is involved in auxin-mediated lateral root formation [68].

A *YAB* family gene (AI782101) was preferentially expressed in Prox and another *YAB* homologue (AK328263) was preferentially expressed in Dis. YAB family proteins act as regulators to establish abaxial cell fates during lateral organ development in Arabidopsis [69]. Recently, a *YAB* homologue gene *SH1* and its homologue genes were identified to regulate seed shattering in cereal species, including sorghum, rice and maize [20].

### Phytohormone-related genes with distinct expression patterns between Prox and Dis

The genes expressed at higher levels in Dis than in Prox included several homologues of genes regulating phytohormone activities (Table 3). For example, BW692346 encodes a homologue of GH3.1, which mediates auxin conjugation [48]; BP875651 and AK247718 encodes homologues of HOOKLESS1 (HLS1), which serves as an integrator of ethylene, auxin, and light signaling pathways in differential cell elongation in Arabidopsis hypocotyls [70]. *SlACO5* encodes a 1-amino-cyclopropane-1-carboxylic acid oxidase (ACO), which catalyzes ethylene biosynthesis and BG130984 and TA49618_4081 are homologues of *CYTOKININ OXIDASE3*, which encodes a cytokinin oxidase that degrades cytokinins. We also found two phytohormone-related genes that are expressed preferentially in Prox, *LIPOXYGENASE A* (*LOXA*) and *Root System Inducible1* (*RSI-1*) (Table 2). *LOXA* encodes a lipoxygenase of the 9-LOX pathway and is proposed to function in biosynthesis of oxylipins [71]. Products of the 9-LOX pathway share similar biological functions with JAs in defense responses [72,73]. *RSI-1* is a member of the *GASA* (for *GA-stimulated Arabidopsis*) gene family, which includes regulators involved in cell and organ elongation [74]; *RSI-1* has been identified as a regulator of lateral root development [75]

### Spatiotemporal expression of *Bl, GOB, Ls* and *LeWUS* in response to an abscission signal

We previously showed that *Bl*, *GOB*, *Ls* and *LeWUS* are preferentially expressed in anthesis pedicel AZs under the regulation of *MC* and *JOINTLESS*. Here we carried out a more detailed analysis of their expression patterns. First, we analyzed their expression during pedicel development from 5 days before anthesis to 3 weeks after anthesis by quantitative RT-PCR (Figure 2A). A substantial amount of *Bl* expression was detected from 5 days before anthesis and the expression reached its maximal level at 2 days after anthesis, while the level was markedly decreased during the fruit growing stage. The highest expression of *GOB* was detected at 3 days before anthesis and then the expression level gradually decreased. Expression of *LeWUS* and *Ls* reached their peak at anthesis and then decreased gradually, similar to that of *GOB.* Unlike the other three genes, expression of *LeWUS* increased again during the fruit growing stage and its highest expression was detected at 3 weeks after anthesis. Next, we examined the effects of a flower abscission signal on the transcript levels of the four genes (Figure 2B). Abscission of the pedicel AZ is stimulated by removing the flower from the pedicel. Following this treatment, 10% and 36% of pedicels were abscised after one and two days, respectively (Additional file 8). After removing flowers, the expression of *GOB*, *Ls* and *LeWUS* at the pedicel immediately declined within 12 hours, but *Bl* expression increased more than 5-fold after the treatment. Subsequently, to identify the cells transcribing the 4 transcription factor genes, AZ regions at anthesis were examined by *in situ* hybridization. Serial sections of the AZ regions were prepared and hybridized with the antisense probes for *Bl*, *GOB*, *Ls* and *LeWUS.* As shown in Figure 2C, mRNAs of *Bl*, *GOB*, *Ls* and *LeWUS* were detected in the same cells; these cells were arranged as several lines within the vascular tissue between the cortex and central parenchyma. When the hybridizations were conducted with sense probes as negative controls, we detected no specific hybridization signals (Additional file 9). The patterns of *Bl*, *GOB*, *Ls* and *LeWUS* were similar to that of *TAPG4* (Additional file 9), whose expression is reportedly detected around the vascular cells within the AZ tissue of the tomato pedicel [53].

## Discussion

### Several pathways in anthesis pedicel AZs may be shared with the regulation of shoot apical meristems (SAMs)

Our analyses of gene expression in anthesis pedicels revealed distinct expression patterns in the AZ compared to other pedicel tissues, Prox and Dis regions. We previously showed that the transcription factors regulating shoot meristem maintenance and lateral shoot development, *LeWUS*, *Bl*, *GOB* and *Ls*, are expressed in anthesis pedicel AZs [10]. In addition to these four genes, here we identified several homologues of transcription factor genes that were identified to regulate meristem activities. *Tkn3* and *BL4*, both of which were up-regulated in AZ, encode homologues of Arabidopsis KNOX and BELL family transcription factors, respectively, which form a heterodimer required for SAM function [76]. Moreover, our analyses also revealed AZ specific up-regulation of *OVATE*, the homologue of which binds to the KNOX-BELL heterodimer complex and modulates activity of the complex [77]. In addition, another transcription pathway found in the SAM may also be activated in anthesis pedicels. Arabidopsis *YAB* genes, which are expressed in abaxial domains of leaves, promote stem cell activity in the meristem through the activity of *LATERAL SUPPRESSOR* (*LAS*), which is expressed at the boundary of the leaf organ primordium and SAM central zone [78,79]. Our analyses found that the *LAS* homologue *Ls* was expressed in the AZ and the *YAB* homologues were expressed outside of the AZ, in the Prox and Dis regions (Table 1, Table 2 and Table 3), suggesting the similarity of the spatial expression patterns of the *LAS* and *YAB* family genes between pedicels and shoot apices. This evidence supports our proposal that transcriptional regulation occurring in AZs is shared with the regulation found in SAMs. If so, how does this common regulation act on these dissimilar tissues and what cell activities does it regulate? Observations of pedicel AZs showed that small cells with densely packed cytoplasm are arranged within AZs [34,80] and these cells are assumed to be maintained in an undifferentiated state [3]. Therefore, one plausible explanation is that the common regulatory system in pedicel AZs and SAMs may serve to maintain small, undifferentiated cells in both tissues. This hypothesis is supported by the fact that *WUS* and *KNOX*, with homologues that were up-regulated in pedicel AZs, are the key genes regulating maintenance of undifferentiated meristem cells [81-83]. Moreover, a recent investigation showed that KNOX family genes determine the timing of floral organ abscission via regulation of the size and proliferation of the AZ cells [36]. In addition, pedicel AZs can develop adventitious shoots, although the event is rare [10], also supporting the possibility that pedicel AZs contain cells with features similar to SAM cells.

### Involvement of *LeWUS*, *Bl*, *GOB* and *Ls* in regulation of competency to respond to abscission-promoting signaling

Here we analyzed expression specificities of *LeWUS*, *Bl*, *GOB* and *Ls* in pedicels to unveil the functions of these AZ-specific genes in abscission. Expression patterns of these 4 genes were remarkably altered by an abscission-initiating stimulus; transcript levels of *LeWUS*, *GOB* and *Ls* declined significantly and *Bl* was substantially up-regulated (Figure 2B). The alternations were significantly different from the expression pattern changes that occur in normal tissue development (Figure 2A). Thus, these four transcription factor genes are likely to be involved in the regulation of the onset of abscission. The expression patterns suggest that *Bl* may be a positive regulator of abscission whereas other three may be negative regulators. During normal tissue development, the expression peaks of these four genes occurred within a few days before and after anthesis (Figure 2A). The developmental stage appears to be critical to determine whether the organ is abscised or transited to the growth phase. Therefore, the tissue may require high level expression of positive and negative regulators to respond immediately to either of the fates. If the tissue is to be abscised, the positive regulators would be up-regulated and the negative ones down-regulated. Alternatively, if the tissue is to transit to the growth phase, the high level expression of both regulators may not be required, probably because abscission is suppressed by the stable supply of the abscission inhibitor auxin from the fruit.

The difference in responses between *Bl* and the other 3 genes was unexpected because the AZ specific expression of all these genes is simultaneously regulated by MC and JOINTLESS [10], and a previous study proposed that *REGULATOR OF AXILLARY MERISTEM* (*RAX*)*, CUP-SHAPED COTYLEDON* (*CUC*) and *LAS*, which are Arabidopsis homologues of *Bl*, *GOB* and *Ls*, are positive regulators that compose a transcription cascade in the axillary meristem [84]. A converse hypothesis, that *RAX* and *LAS* are elements of two independent transcription pathways, was also proposed [85]. The expression of *Bl, LeWUS*, *GOB* and *Ls* in AZ may be regulated by dual mechanisms, in which a common regulatory factor may induce these four genes before the onset of anthesis, and once an abscission signal is provided, different transcriptional regulators may up-regulate *Bl* or down-regulate the other three genes.

The expression properties of *LeWUS* and *GOB* indicate a probable explanation for the physiological changes in pedicel AZ cells during abscission*.* Histological analysis of the tomato pedicel AZ revealed that the separation zone cells that remain small before abscission do enlarge just after the onset of abscission [86]. It has been proposed that the enlargement of AZ cells at the onset of abscission produces a force that ruptures the separation zone [36,87]. As described above, *WUS* functions to maintain cells in an undifferentiated state in SAMs; *CUC*, a homologue of *GOB,* also acts to keep cell size small [83,88]. Therefore, the reduced expression of *GOB* and *LeWUS* caused by an abscission signal may result in the enlargement of the separation zone cells for the onset of abscission. Of the four genes, only *LeWUS* showed increased expression at 3 weeks after anthesis. Because the separation zone cells increase until the mature green fruit stage [80], increasing activity of *LeWUS* may be required to maintain the physiology of the cells.

As shown in Figure 2C, *LeWUS*, *Bl*, *GOB* and *Ls* were all expressed in the vascular cells of the pedicel, but not in the small cells at the separation zone. In SAMs, *WUS* regulates stem cell activity and is expressed in cells underneath the stem cells, but not in the stem cells themselves [83]. *RAX*, *CUC* and *LAS*, the homologues of *Bl*, *GOB* and *Ls*, respectively, are expressed in a boundary region between the SAM and leaf primordium [84,85,89]. If separation zone cells in the pedicel AZs possess similar properties with SAMs as described above, the expression of *LeWUS*, *Bl*, *GOB* and *Ls* outside of the separation zone cells is consistent with the observation in SAMs. These four genes may regulate activities of the separation zone cells in AZs through non-cell autonomous mechanisms similar to the regulation of SAM activity by *WUS* or *LAS*[78,90]. Although the functions of these 4 genes in pedicel AZs is still unclear, our expression analyses suggest that these four genes make significant contributions to the response to the onset of the abscission. Further investigation will provide key insights into the functions of these transcription factors in the regulation of abscission.

### An auxin gradient may be formed in the pedicel and affect gene expression

Several lines of evidence indicate that auxin and ethylene are critical factors that regulate the onset of abscission, and auxin plays a role in maintenance of flower or fruit attachment to the plants [1,22]. Our results revealed that expression patterns of genes that are involved in auxin signaling and auxin homeostasis were obviously different in the three pedicel regions, AZ, Prox and Dis. Homologues of the genes for IAA amide synthases DFL1/GH3.6 (AK319847) and GH3.1 (BW692346) were preferentially expressed in AZ and Dis, respectively, but no homologue was found in the genes preferentially expressed in the Prox region. IAA amide synthase genes are induced in auxin-abundant tissues and the gene products inactivate IAA to control auxin homeostasis [91], suggesting that the auxin concentrations in the Dis and AZ regions at anthesis are higher than that in the Prox region. Meanwhile, genes preferentially expressed in the AZ included a homologue of the gene for MES1 (AK328818), which can convert an inactive form of IAA, IAA-methyl ester (MeIAA), into the active form, IAA [49]. These results suggest that the level of active IAA in AZ at anthesis may be fine-tuned in a complex manner. These opposite reactions, namely activation and inactivation of IAA, may occur in different cell-groups within the AZ to maintain its pre-abscission status. We also found a homologue of *IAA29*, an auxin-inducible transcription factor gene [66], in the genes preferentially expressed in the Dis region (AK319758; Table 3). The expression of the *IAA29* homologue was the highest in the Dis region and gradually decreased toward Prox (Additional file 10). In addition, several genes that may play roles in auxin signaling, such as homologues of *MYB77* (BG126724) and *NAC1* (TC202847)*,* had more abundant transcripts in Dis than in Prox (Table 3). These results also support the possibility that an auxin gradient is formed in the pedicel tissues. In contrast, the transcript level of the auxin response factor *ARF9* homologue was significantly higher in Prox (Table 2 and Additional file 10). Generally, transcription factor activities of ARFs are modulated by post-transcriptional regulation; ARF activity is inhibited by conjugation of AUX/IAA proteins and the ARFs are activated when the auxin level in the cell is elevated and the increased auxin induces degradation of the conjugated AUX/IAAs [92]. Although it is unknown whether the transcript level of the *ARF9* homologue in Prox is regulated by auxin concentration, the high level expression in Prox suggests that the Prox region may show a response to auxin that is distinct from the AZ and Dis regions. These different responses to auxin are likely to reflect the different gene expression patterns in each tissue. Because the auxin level determines the timing of onset of pedicel abscission [27], the auxin signaling genes found in this study, especially those expressed in AZ and Dis region, may be involved in preventing the onset of abscission.

### MADS box proteins may be key factors that determine the identities of pedicel regions

Our results showed that despite their similar external appearances, the Prox and Dis regions in anthesis pedicels have distinct expression profiles. In particular, the Dis region is characterized by the specific expression of the genes related to floral organ development such as homologues of *AP2* (*SlAP2a*) and MADS box genes *AGL6* (BI203609), *AP3* (*TAP3*)*, SEP1* (*TM29 and SlMBP21*) and *SEP3* (*TDR5*), suggesting that Dis has floral organ like identity at the gene expression level (Table 3). By contrast, the Prox region cells specifically expressed a homologue of *SOC1* (*SlMBP14*), which encodes another MADS box protein that regulates the transition from vegetative to reproductive growth [62,63]. The region-specific expression of transcription factor genes that determine distinct cell fates suggests that the Prox and Dis tissues have distinct developmental identities.

Our previous study indicated that the MADS box genes *MC* and *JOINTLESS*, which are expressed throughout the pedicel tissues, regulate pedicel AZ development and also induce AZ specific expression of *LeWUS*, *Bl*, *GOB* and *Ls*[10]*.* These observations suggest that MADS box transcription factors may substantially contribute to specification of the identity of the pedicel regions. The MADS box proteins that are expressed in each pedicel region may form region-specific protein complexes and determine the identities of each region, similarly to the quartet model proposed in flower organ identification [93].

## Conclusion

To unveil the transcriptional properties of tomato pedicels at the pre-abscission state, we analyzed the gene expression profiles of three flower pedicel regions, Prox, AZ and Dis. The gene expression data indicated that there are substantial differences between AZ and non-AZ tissues (Prox and Dis), and also between Prox and Dis. In particular, genes involved in auxin activity showed distinct expression patterns in the pedicel regions, suggesting that a gradient of auxin concentration may be formed throughout the pedicel regions and this auxin gradient may be one of the key factors affecting the distinct expression patterns in the pedicel tissues. These auxin-related genes may play a critical role in the regulation of timing of abscission. Various groups of transcription factors were also expressed in a region-specific manner, and of these, different types of MADS-box transcription factor genes were found in different regions. For example, the Dis region cells express the MADS-box genes required for floral organ development, whereas the Prox region cells express another MADS-box gene regulating flowering. Several types of MADS-box protein complexes may form in the respective pedicel regions specifically to regulate gene expression, similarly to the floral quartet model. A couple of transcription factor genes associated with apical or axillary shoot meristem function were found to be expressed preferentially in the AZ, indicating that shoot meristem cells and AZ cells may have common meristematic functions. Finally, the detailed gene expression analyses of *Bl, GOB*, *Ls* and *LeWUS* suggest that these AZ specific transcription factor genes may be key factors responding to an abscission cue, although the regulatory pathways may be different between these four genes; *Bl* may be a positive regulator of abscission and the other three may be negative regulators.

In conclusion, this study identifies multiple differentially expressed factors that may be important in establishing and maintaining the properties of pre-abscission tomato pedicel AZs, and provides insights into the transcriptional regulation of pre-abscission responses. Especially, comparative study of genes commonly expressed in both AZs and SAMs may provide a new aspect of the regulation in the abscission. In addition, the pedicel region specific MADS box transcription factors would be attractive candidates for the regulators determining pedicel tissue identities.

## Methods

### Microarray experiments and data analysis

Expression analyses were performed on total RNAs extracted from tomato (*Solanum lycopersicum*) cv. Ailsa Craig grown in soil in a growth room at 25°C with 16 h day length. Each pedicel region was carefully cut off using a sharp razor blade and subjected to analysis. For hybridization, we used the Agilent Tomato Gene Expression Microarray 44K (Agilent Technologies, Santa Clara CA, USA), which contains probes designed from the EST sequences deposited in three different databases, GeneBank (GB accessions), TIGR (TA accessions) and the Tomato Gene Index (TC and NP accessions). Hybridization and signal detection was performed essentially as described previously [10]. Signal intensities were normalized by the per chip normalization method to the 75th percentile using GeneSpring software version 10.0 (Agilent Technologies). Data from outlier probes were removed if the signal was a non-uniform outlier, or if the signal was a population outlier. Data from three independently prepared samples were evaluated by a one-sample t-test with the log_2_-transformed signal ratios of each probe using MeV v4.6.2 software with the default setting using the options “*p-values based on t-distribution”* and “*Just Alpha (no correction)*” [94]. To annotate tomato ESTs whose sequences were used for microarray probes, we searched the Arabidopsis Information Resource protein database (TAIR9) by BLASTX with the expect value threshold at 0.01. The ESTs for the microarray probes were assigned to the coding sequences (CDS) predicted by ITAG (version 2.3) via BLASTN searches with expect value threshold at 1e-10 on the International Solanaceae Genomics project (SOL) genomics network website (http://solgenomics.net/). The complete microarray data set has been deposited at the Gene Expression Omnibus with accession number GSE39519 [NCBI GEO].

### RT-PCR and quantitative RT-PCR analysis

First strand cDNAs for RT-PCR experiments were synthesized with the PrimeScript 1st strand cDNA Synthesis Kit (Takara Bio Inc., Otsu Shiga, Japan). The RT-PCR was performed using ExTaq polymerase (Takara Bio Inc.) following the manufacturer’s instructions. Quantitative PCR amplification was carried out with the 7300 Real-Time PCR System (Applied Biosystems, Foster City, CA, USA) using THUNDERBIRD SYBR qPCR MIX (TOYOBO, Osaka, Japan). Relative quantification of the expression of each gene was performed using the 2^-ΔΔCT^ method [95]. The *SAND* (SGN-U316474) gene was used as an internal control [96]. Oligonucleotides used for PCR are listed in Additional file 11.

### In situ hybridization

Anthesis flower pedicels were fixed with formalin/acetic acid/alcohol (FAA), embedded in paraffin and then sectioned at 4 μm thickness. Probe hybridization was performed according to the previously described method [97]. Color reactions were performed with NBT/BCIP. A control section was stained with hematoxylin and eosin stain. Each section was counterstained with Kernechtrot stain and mounted with CC/Mount (Sigma-Aldrich). To produce DIG-labeled RNA probes, cDNA fragments were PCR-amplified (oligonucleotides listed in Additional file 11) and cloned into pSPT19 (Roche); DIG-labeled RNAs were synthesized with T7 RNA polymerase.

## Competing interests

The authors declare that they have no competing interests.

## Authors’ contributions

TN and YI designed the research; TN performed the research and analyzed data; MF contributed to the microarray data analysis; YS contributed to the sample preparation; TN and YI wrote the paper. All authors read and approved the final draft.

## Supplementary Material

Additional file 1 Genes up-regulated in AZ compared with Prox. Click here for file

Additional file 2 Genes up-regulated in AZ compared with Dis. Click here for file

Additional file 3 **Validation of the microarray data by RT-PCR assays on genes that exhibited AZ preferential expression by the microarray assays.** Transcript levels of 10 genes in the pedicel regions, AZ, Prox and Dis, were compared for the microarray and RT-PCR assays. The results obtained by the two methods showed good consistency for all the examined genes. As an internal control (Ctrl) for the RT-PCR assays, *SAND* (SGN-U316474) was used [96]. Click here for file

Additional file 4 Genes down-regulated in AZ compared with non-AZ (Prox and Dis). Click here for file

Additional file 5 Genes up-regulated in Prox compared with Dis. Click here for file

Additional file 6 Genes up-regulated in Dis compared with Prox. Click here for file

Additional file 7 **Validation of the microarray data by RT-PCR on genes differentially expressed between Prox and Dis.** Transcript levels of 11 genes in the pedicel regions, AZ, Prox and Dis, were compared with the microarray assays and RT-PCR assays. The results obtained by the two methods showed good consistency for all the examined genes. As an internal control (Ctrl) for the RT-PCR assays, *SAND* (SGN-U316474) was used [96]. Click here for file

Additional file 8 **Frequency of flower pedicel abscission after removal of the flower.** Anthesis flowers were removed from the pedicels to induce abscission and then the number of abscised pedicels was counted. In total, 206 flower pedicels were used for the analysis. Click here for file

Additional file 9 **Expression of *Bl*, *GOB*, *Ls, LeWUS *and *TAPG4 *in the AZ of a tomato flower pedicel at anthesis.** When tissue sections of the AZ of a flower pedicel at anthesis were hybridized with the DIG-labeled antisense probes for *Bl*, *GOB*, *Ls LeWUS* and *TAPG4,* the hybridization signals were detected in tube-like vascular cells but no specific signals were detected when their sense probe were examined. The signals for each gene are indicated by arrowheads. Click here for file

Additional file 10 **The expression patterns of TA50096_4081 (an *ARF9 *homologue) and AK319758 (an *IAA29 *homologue) in tomato flower anthesis pedicels.** The transcript level of AK319758 was the highest in Dis and decreased gradually toward Prox; TA50096_4081 was significantly higher in Prox compared to Dis. The expression signal intensity was detected by microarray assays and error bars indicate standard deviation of biological triplicates for the assay. Click here for file

Additional file 11 Sequences of primers used in this study. Click here for file
