# Supplementary figures and images for: Expression profiling of tomato pre-abscission pedicels provides insights into abscission zone properties including competence to respond to abscission signals
Source: BMC Plant Biol. 2013 Mar 9;13:40. doi: 10.1186/1471-2229-13-40 (PMC3600680; doi:10.1186/1471-2229-13-40)

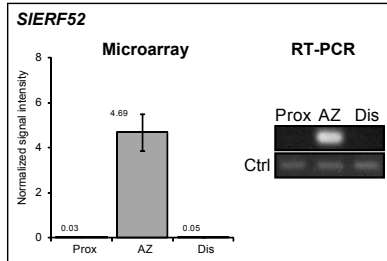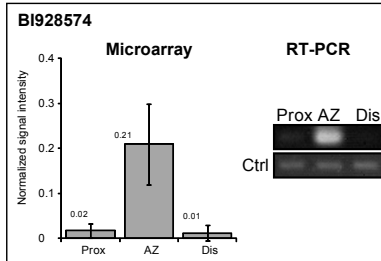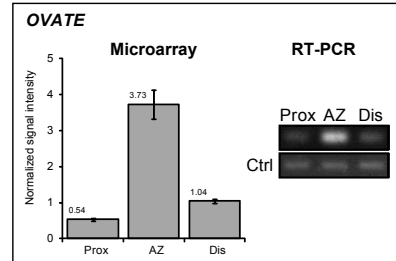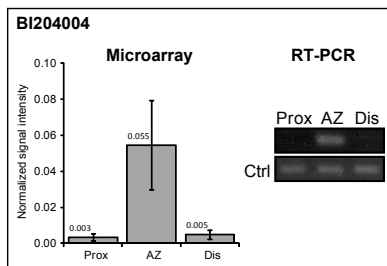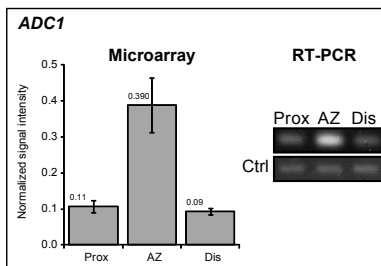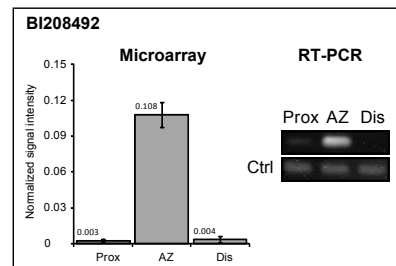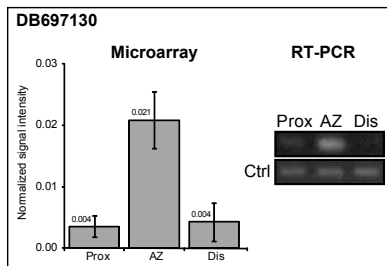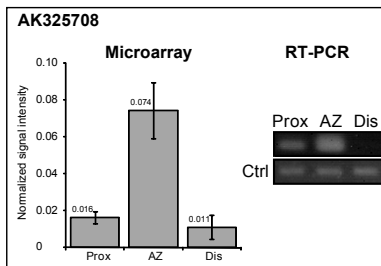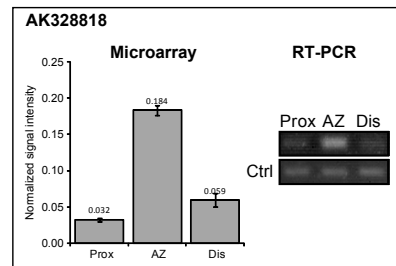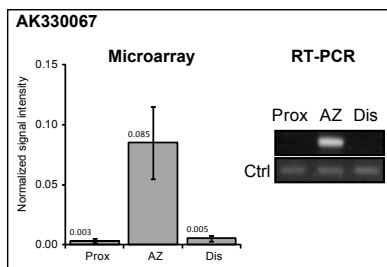

Supplement: Additional file 3 — Validation of the microarray data by RT-PCR assays on genes that exhibited AZ preferential expression by the microarray assays. Transcript levels of 10 genes in the pedicel regions, AZ, Prox and Dis, were compared for the microarray and RT-PCR assays. The results obtained by the two methods showed good consistency for all the examined genes. As an internal control (Ctrl) for the RT-PCR assays, SAND (SGN-U316474) was used [96]. [file 1471-2229-13-40-S3.pdf]

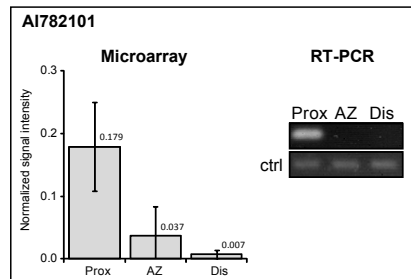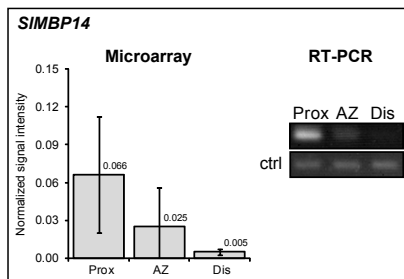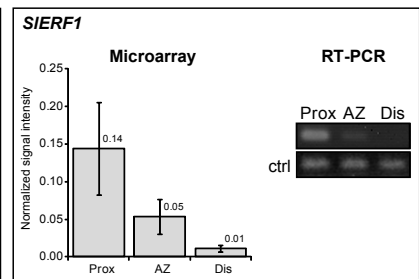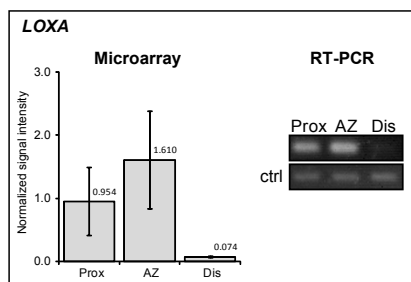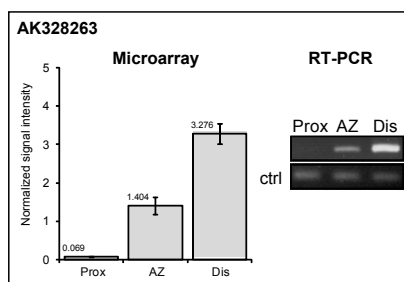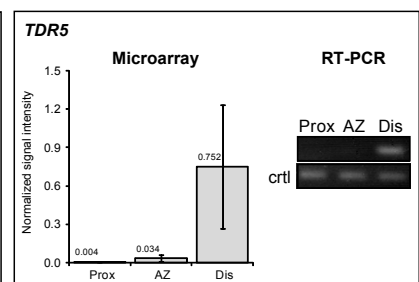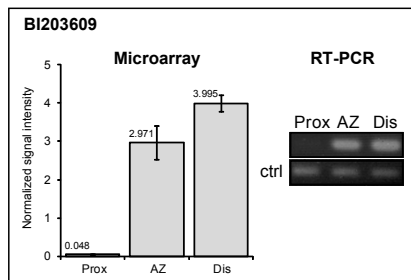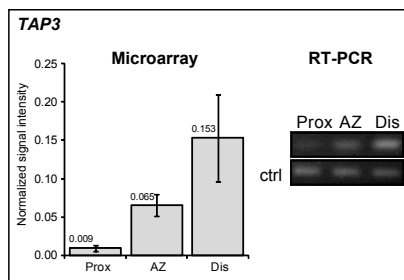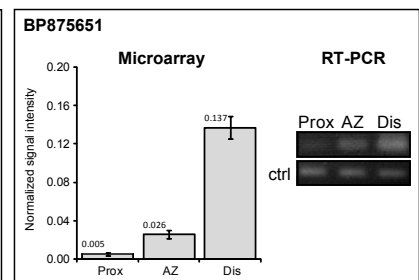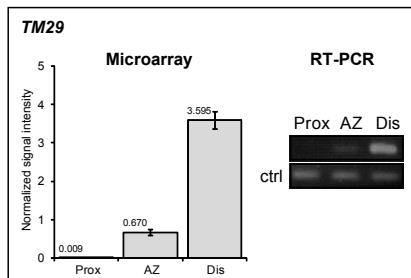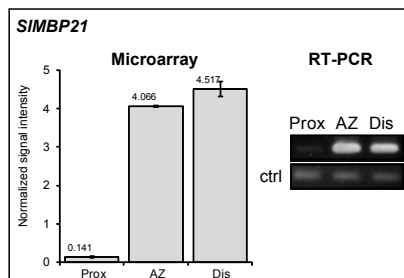

Supplement: Additional file 7 — Validation of the microarray data by RT-PCR on genes differentially expressed between Prox and Dis. Transcript levels of 11 genes in the pedicel regions, AZ, Prox and Dis, were compared with the microarray assays and RT-PCR assays. The results obtained by the two methods showed good consistency for all the examined genes. As an internal control (Ctrl) for the RT-PCR assays, SAND (SGN-U316474) was used [96]. [file 1471-2229-13-40-S7.pdf]

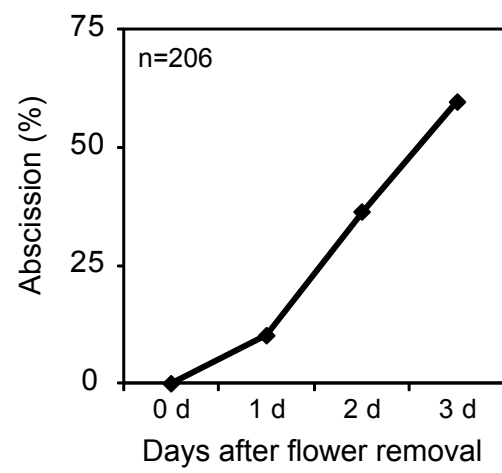

Supplement: Additional file 8 — Frequency of flower pedicel abscission after removal of the flower. Anthesis flowers were removed from the pedicels to induce abscission and then the number of abscised pedicels was counted. In total, 206 flower pedicels were used for the analysis. [file 1471-2229-13-40-S8.pdf]

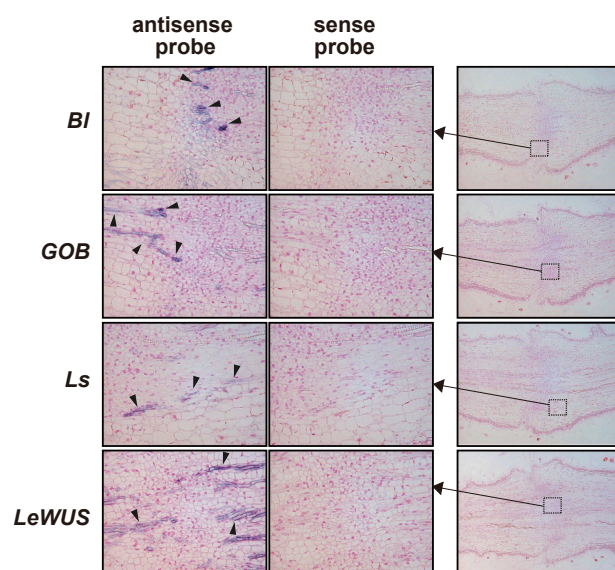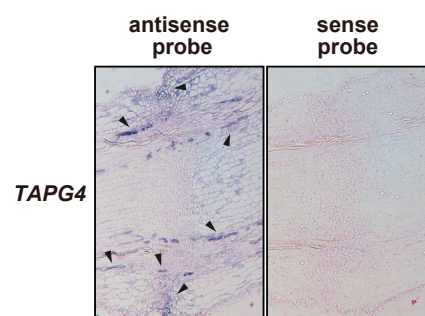

Supplement: Additional file 9 — Expression of Bl, GOB, Ls, LeWUS and TAPG4 in the AZ of a tomato flower pedicel at anthesis. When tissue sections of the AZ of a flower pedicel at anthesis were hybridized with the DIG-labeled antisense probes for Bl, GOB, Ls LeWUS and TAPG4, the hybridization signals were detected in tube-like vascular cells but no specific signals were detected when their sense probe were examined. The signals for each gene are indicated by arrowheads. [file 1471-2229-13-40-S9.pdf]

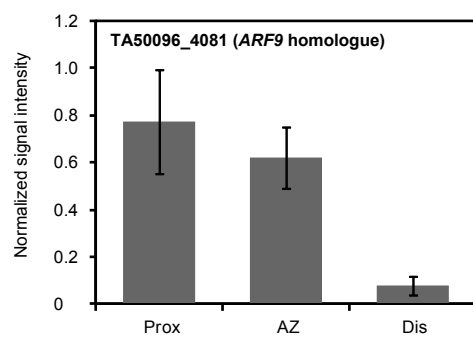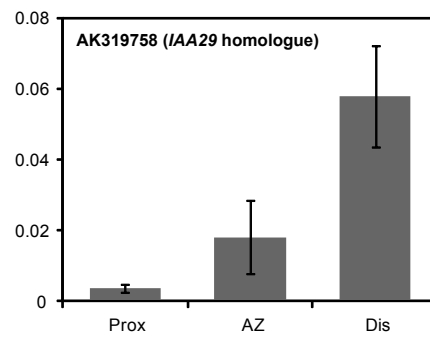

Supplement: Additional file 10 — The expression patterns of TA50096_4081 (an ARF9 homologue) and AK319758 (an IAA29 homologue) in tomato flower anthesis pedicels. The transcript level of AK319758 was the highest in Dis and decreased gradually toward Prox; TA50096_4081 was significantly higher in Prox compared to Dis. The expression signal intensity was detected by microarray assays and error bars indicate standard deviation of biological triplicates for the assay. [file 1471-2229-13-40-S10.pdf]
